# Monodispersed mesoscopic star-shaped gold particles via silver-ion-assisted multi-directional growth for highly sensitive SERS-active substrates

**DOI:** 10.1186/s40580-024-00435-4

**Published:** 2024-07-04

**Authors:** Sumin Kim, Sunghoon Yoo, Dong Hwan Nam, Hayoung Kim, Jason H. Hafner, Seunghyun Lee

**Affiliations:** 1 Department of Applied Chemistry, Hanyang University ERICA, Ansan, 15588 Republic of Korea; 2 Department of Chemical and Molecular Engineering, Hanyang University ERICA, Ansan, 15588 Republic of Korea; 3 Center for Bionano Intelligence Education and Research, Hanyang University ERICA, Ansan, 15588 Republic of Korea; 4 Department of Physics and Astronomy, Rice University, 6100 Main St, Houston, TX 77005- 1827 USA

**Keywords:** Plasmonic, Gold particles, LSPR, Ag ion, Reducing agent, SERS

## Abstract

Surface-enhanced Raman scattering (SERS) exploits localized surface plasmon resonances in metallic nanostructures to significantly amplify Raman signals and perform ultrasensitive analyses. A critical factor for SERS-based analysis systems is the formation of numerous electromagnetic hot spots within the nanostructures, which represent regions with highly concentrated fields emerging from excited localized surface plasmons. These intense hotspot fields can amplify the Raman signal by several orders of magnitude, facilitating analyte detection at extremely low concentrations and highly sensitive molecular identification at the single-nanoparticle level. In this study, mesoscopic star-shaped gold particles (gold mesostars) were synthesized using a three-step seed-mediated growth approach coupled with the addition of silver ions. Our study confirms the successful synthesis of gold mesostars with numerous sharp tips via the multi-directional growth effect induced by the underpotential deposition of silver adatoms (AgUPD) onto the gold surfaces. The AgUPD process affects the nanocrystal growth kinetics of the noble metal and its morphological evolution, thereby leading to intricate nanostructures with high-index facets and protruding tips or branches. Mesoscopic gold particles with a distinctive star-like morphology featuring multiple sharp projections from the central core were synthesized by exploiting this phenomenon. Sharp tips of the gold mesostars facilitate intense localized electromagnetic fields, which result in strong SERS enhancements at the single-particle level. Electromagnetic fields can be further enhanced by interparticle hot spots in addition to the intraparticle local field enhancements when arranged in multilayered arrays on substrates, rendering these arrays as highly efficient SERS-active substrates with improved sensitivity. Evaluation using Raman-tagged analytes revealed a higher SERS signal intensity compared to that of individual mesostars because of interparticle hot spots enhancements. These substrates enabled analyte detection at a concentration of 10^− 9^ M, demonstrating their remarkable sensitivity for trace analysis applications.

**Supplementary Information:**

The online version contains supplementary material available at 10.1186/s40580-024-00435-4.

## Introduction

Plasmonic metal nanoparticles (NPs) have gained considerable research interest because of their unique localized surface plasmon resonance (LSPR) optical properties. The LSPR can be attributed to the collective oscillation of free electrons on the NP surface caused by the interaction with incident light at specific wavelengths. This phenomenon induces strong visible and near-infrared scattering/absorption peaks, as well as highly concentrated electromagnetic fields localized on the particle surface. The optical characteristics of plasmonic NPs are strongly dependent on their size and shape, driving extensive efforts towards synthesizing monodisperse and shape-controlled NPs with high yields [1–4]. The LSPR serves as a powerful platform for detecting molecular interactions occurring near the NP surface because the resonant frequency of the localized electromagnetic field is sensitive to the local refractive-index environment around the NP. The enhanced localized field effect is prominent at the sharp edges and tips of the anisotropic NPs [5–8]. Given their unique optical properties, AuNPs are widely applied to bioanalysis [9, 10], molecular imaging [11], photodynamic therapy [12], drug delivery [13], biomedical applications [14], and biosensors [15–17] based on surface-enhanced Raman scattering (SERS). Among these, SERS-based nanobiosensors offer several advantages, including the ability to detect analytes at the single-particle level, analyze extremely small sample volumes, and achieve high sensitivity and specificity. Uniformly generating hot spots that amplify the Raman signal generated by the strong electromagnetic fields formed on the particle surfaces is critical for enhancing the SERS efficiency of AuNPs. The local electromagnetic field can be enhanced further when the distance between AuNPs with localized strong electric fields is sufficiently small, leading to the formation of plasmonic hot spots with field intensities surpassing those of individual particles [18, 19]. Consequently, the SERS efficiency can be improved by synthesizing and strategically arranging NPs with structures that can generate numerous hot spots.

The NPs with distinct and sharp geometries have attracted considerable attention because of their ability to generate hot spots characterized by enhanced effects when local electromagnetic field enhancement focuses on sharp edges [5, 20–23]. Consequently, the strategic synthesis of anisotropic AuNPs specifically designed with numerous sharp tips has immense potential for improving SERS signal amplification and enhancing detection sensitivity. However, obtaining uniform mesoscale gold particles presents a significant challenge. In addition, previous studies have reported the synthesis of star-shaped Au NPs with a spherical core ~ 100 nm in diameter and multiple protruding tip-shaped nanostructures.

In this study, we propose a novel approach for synthesizing gold mesostars characterized by their complex structure and numerous sharp protrusions on the surface. The primary objective is developing gold mesostars for use as highly sensitive SERS-active substrates, contributing to the application of molecular-sensing techniques with high sensitivity and accuracy. Gold mesostars offer intriguing prospects as SERS substrates because of the emergence of LSPR peaks at multiple wavelengths, which result from plasmon hybridization facilitated by their intricate surface structure. The presence of sharp peaks also induces localized field enhancement effects [24]. In contrast to the previous reports [25–29], our group developed the high-yield synthesis of gold mesostars with numerous tips within a single particle using a three-step seed-mediated growth method. In this method, silver ions were added during the last step of the synthesis to produce gold mesostars. The added Ag ions were deposited on a specific surface of gold through underpotential deposition (UPD). The underpotential deposition of silver (AgUPD) can control the final shape of the NPs when silver supports the growth of gold particles [30, 31]. AgUPD refers to the reduction of Ag^+^ to Ag^0^ on a metal substrate with a surface potential lower than the bulk reduction potential of Ag^+^. Ag is preferentially deposited on the {110} side of the gold surface, which has a higher surface energy compared to that of the {100} or {111} facets, resulting in the formation of a silver monolayer that inhibits further gold growth on the {110} side. Transmission electron microscopy (TEM), fast Fourier transform (FFT), and selected area electron diffraction (SAED) patterns confirm that the tips of the star-shaped particles are bound by the (111) plane. Our observations indicate that the presence of Ag ions facilitates multi-directional growth, and by controlling the Ag ion concentration, particles with sharp tips can be synthesized, achieving strong localized field enhancement effects. We compared the morphologies of the synthesized particles and identified the conditions that yielded particles with the smallest tip curvatures and mesoscale dimensions by optimizing the amounts of silver nitrate and ascorbic acid. Subsequently, the synthesized particles were subjected to SERS measurements at the single-particle level for evaluating the extent of electromagnetic field enhancement arising from the tip curvature and particle shape. A significantly stronger SERS signal was observed when SERS-active substrates were fabricated using the optimized gold mesostars; this can be attributed to the synergistic effects of electromagnetic enhancements from the sharp particle tips and interparticle hot spots. These substrates enabled the detection of Raman-tagged molecules at concentrations as low as 10^− 9^ M, confirming the potential of gold mesostar-based SERS-active substrates for ultrasensitive molecular sensing applications.

## Experimental method

### Materials

Hexadecyltrimethylammonium bromide (CTAB, ≥ 99%), L-Ascorbic acid (AA, ≥ 99.0%), sodium borohydride (≥ 98.0%), gold (III) chloride trihydrate (HAuCl_4_, ≥ 99.9%), nitic acid (0.1 M), and silver nitrate (AgNO_3_, ≥ 99.0%) were purchased from Sigma-Aldrich. Malachite Green isothiocyanate (MGITC) was purchased from Thermo Scientific.

### Synthesis of au seeds

Gold seeds were prepared with 9.75 mL of 50 mM CTAB and 0.25 mL of 10 mM HAuCl_4_ by brief and gentle inversion. Next, 0.35 mL of a freshly prepared ice-cold 10 mM NaBH_4_ solution was added, followed by vigorous mixing for 2 min. The seed solution was pale brown in color.

### Synthesis of gold Mesostars

Three different growth solutions based on one basic growth solution prepared as 3.465 g of CTAB in 99 mL of distilled water containing 1mL of 25 mM HAuCl_4_ stock solution were prepared for synthesizing gold mesostars. The following three solutions were prepared in three separate glass vials, where each vial was individually tagged as A, B, or C. Subsequently, 0.025 mL of the 100 mM AA solution was added to 4.5 mL of the basic growth solution in each glass vial (A and B). In a third glass vial (C), 0.3 mL of 100 mM nitric acid and 0.25 mL of 100 mM AA were added to 45 mL of the basic growth solution. Next, 0.4 mL of the Au seeding solution was added to solution A, and immediately, 0.4 mL of this mixed solution A was added to solution B. Immediately thereafter, 4 mL of the solution from mixed solution B was added to solution C. All solutions were well mixed by gentle inversion for a few seconds in each step. Finally, 0.5 mL of 10 mM AgNO_3_ (0.5 mL) was added to solution C. The solution was maintained at 40 °C for two days. During this time, the color of the solution remained clear; however, after the reaction was complete, gold mesostars with a pale peach-orange color precipitated to the bottom. A colorless solution containing excess CTAB was decanted to collect the gold mesostars, and the particles were redispersed in DI water.

### Preparation of the SERS substrate

The gold mesostars solution (10 µL) was dropped on a silicon wafer and dried in an oven for 12 h. After drying, the Au substrate was incubated in a 1 µM Raman tag molecule (MGITC) solution for 12 h. Subsequently, the substrate was rinsed with DI water, and after drying under nitrogen flow, the substrate was ready for Raman studies. The limit of detection was measured by adjusting the Raman tag molecule concentration to 10^− 6^, 10^− 7^, 10^− 8^, and 10^− 9^ M, although all other processes were the same.

### Instrumentation

Scanning electron microscope (SEM) images were obtained using a Hitachi S-4800 instrument. Field-emission transmission electron microscopy (FE-TEM) images were obtained using JEOL JEM-2100 F and JEM-F200. The ultraviolet-visible-near-infrared (UV-Vis-NIR) absorption spectra were measured using a JASCO V-770 spectrophotometer. SERS signals were measured using confocal micro-Raman spectroscopy with HEDA and WEVE, and single-particle scattering spectra were obtained using dark-field microscopy with HEDA and WEVE.

## Results and discussion

**Scheme 1:**
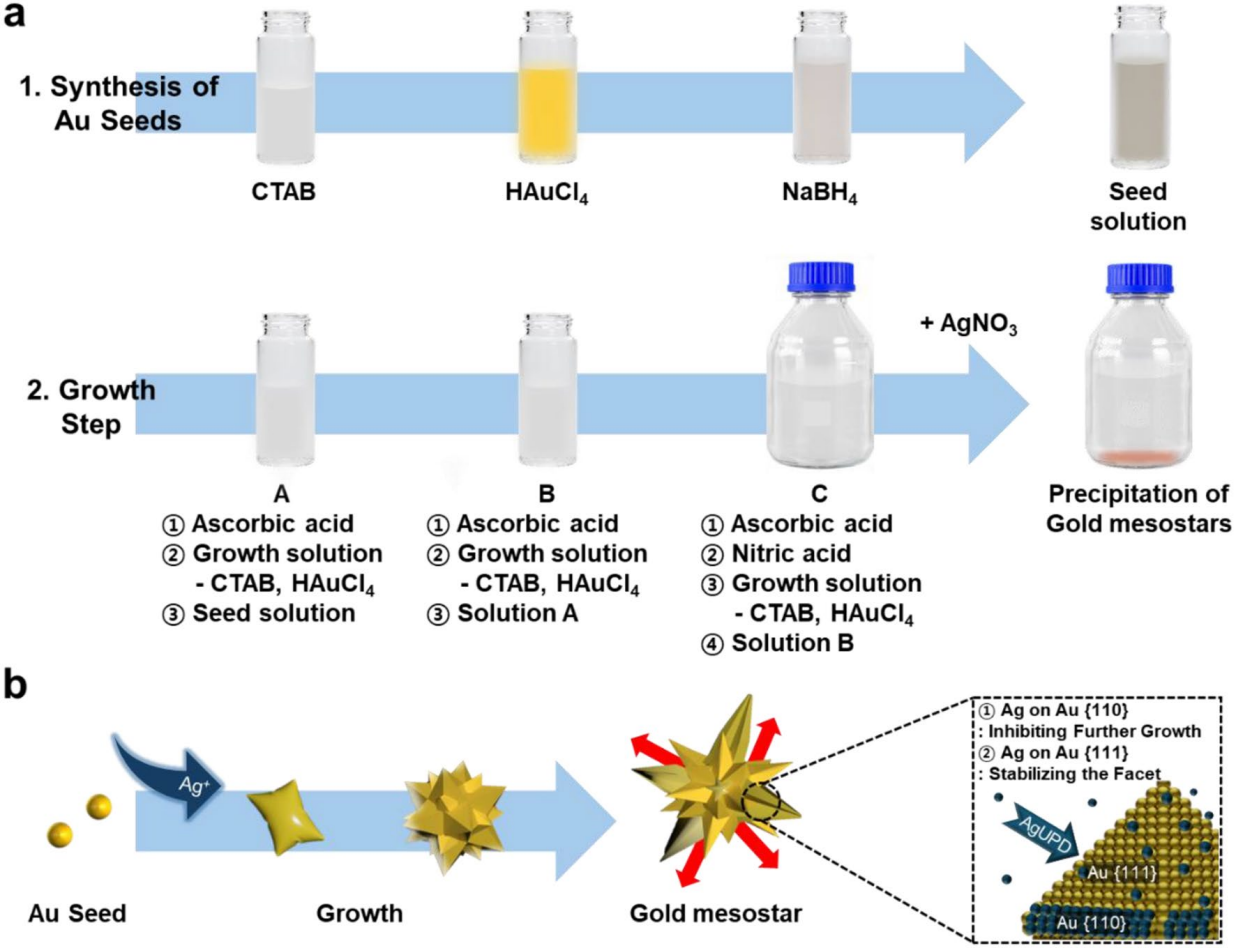
(**a**) A three-step seed-mediated growth synthesis scheme for gold mesostars. (**b**) A schematic diagram showing the effects of AgUPD

**Fig. 1:**
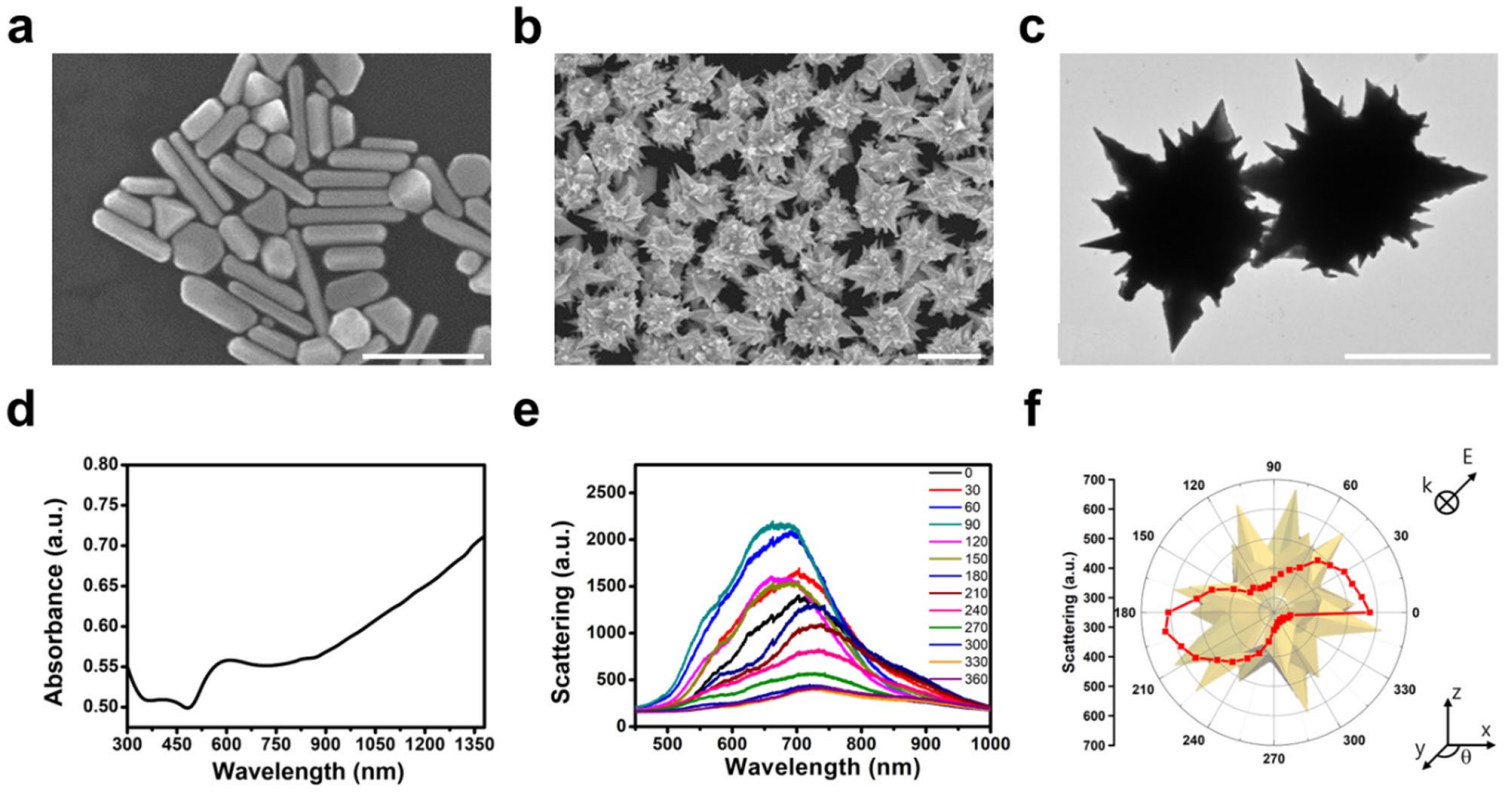
SEM images of gold particles in the (**a**) absence and (**b**) presence of silver ions. (**c**) TEM image of gold mesostars. All scale bars represent 1 μm. (**d**) Absorbance spectra of gold mesostars. (**e**) The scattering spectrum of a single particle (polarized by 0 to 360°). (**f**) Polar plot of angular-dependent scattering

Gold mesostars were successfully synthesized by adding AgNO_3_ via a three-step seed-mediated growth method (Scheme. 1a). The Ag ions are believed to influence and control the final shape of AuNPs when synthesizing them through a property known as AgUPD (Scheme. 1b). If AgNO_3_ is not added in the last step of the synthesis, the nanoparticle growth cannot be oriented, which results in synthesizing inhomogeneous particles such as gold nanorods and plates instead of gold mesostars (Fig. 1a). However, multifaceted anisotropic gold mesostars were synthesized when AgNO_3_ is added, and homogeneous, high-yield particles are obtained using the seed-mediated three-step growth method. The SEM and TEM images in Fig. 1b and c show that the synthesized particles have sharp tips with multifaceted growth on the surface. The absorption of the synthesized gold mesostars is confirmed by the UV-Vis-NIR spectrum, which shows a broad peak in the region above 1200 nm because of the plasmon hybridization of numerous tips (Fig. 1d) [24]. The scattering spectrum was measured by adjusting the angle of light irradiation to analyze the optical properties of a single gold mesostar (Fig. 1e and f). The scattered light was polarized and measured using a polarizing filter for determining the degree of scattering caused by the particle structure. Accurately observing the spectrum of the tip as a function of the angle is difficult because the complex surface structure of the gold mesostar causes multiple peaks to be located at similar wavelengths, resulting in overlapping spectra. Different peaks form or disappear as the polarizing filter of the analyzer is rotated because the scattered light is plasmonically resonated by each tip at its unique angle, and the gold mesostar exhibits strong plasmon resonance characteristics owing to the numerous tips on the particle surface [24, 29].

We confirmed that Ag ions play a decisive role in the synthesis of gold mesostars. We systematically adjusted the AgNO_3_ volume to investigate the morphological changes induced by AgNO_3_. This silver-assisted growth method was facilitated by AgUPD on specific crystal facets of gold. The differences in the work functions between Ag and Au (110), (100), and (111) planes are 0.85, 0.83, and 0.57 eV, respectively. Consequently, the adsorption of Ag ions is preferred in the order of Au (110), (100), and (111) planes. In other words, the deposition of Ag ions predominantly occurs on the {110} facets, inhibiting the growth of Au along the {110} facet, thereby promoting anisotropic growth. When particles grow along the twinning axis, the Au {100} side forms step structures rather than a flat structure, such as a gold nanorod, and the adsorbed Ag ions stabilize this structure, thereby forming bipyramids [31]. However, the interaction between the AuCl_2_ and CTAB complex and the gold seeds increases when the gold concentration in the growth solution is high, which results in the growth of multibranched particles instead of bipyramids. [35]

**Fig. 2:**
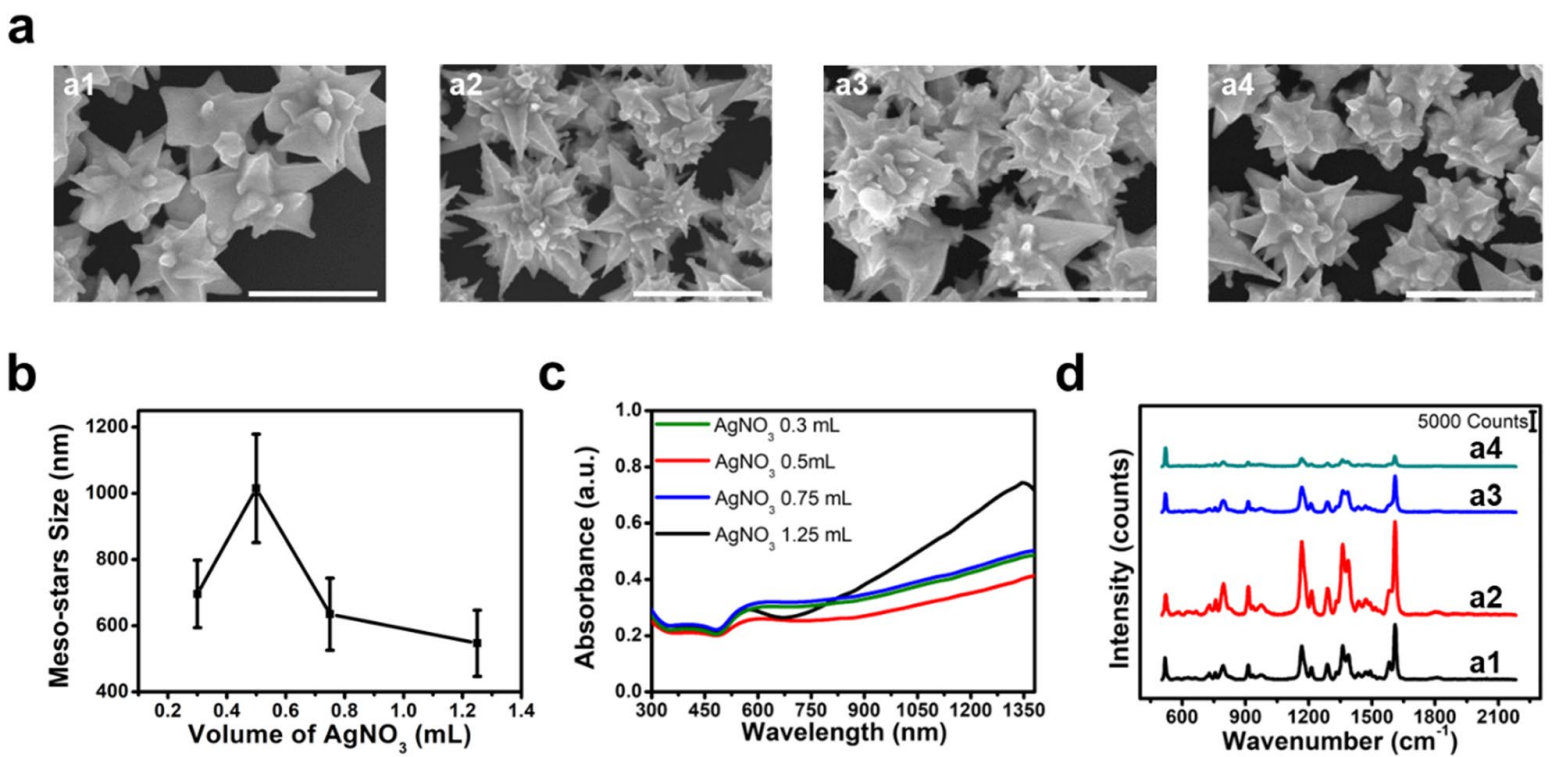
(**a**) SEM images of gold mesostars synthesized through the adjustment of AgNO_3_ volumes (a1–a4 correspond to AgNO_3_ volumes of 0.3, 0.5, 0.75, and 1.25 mL, respectively). All scale bars represent 1 μm. (**b**) Size variations of gold mesostars. (**c**) UV-Vis-NIR spectra of gold mesostars. (**d**) SERS signals according to AgNO_3_ volume

Irregular particles, including gold nanorods and plates, are obtained instead of gold mesostars when AgNO_3_ is not added during the final synthesis step. The volume of AgNO_3_ was systematically varied to investigate the effect of the Ag ion concentration on particle formation. Increasing the AgNO_3_ volume up to 0.5 mL resulted in the formation of mesoscale particles with sharp tips; however, beyond this volume, excess AgNO_3_ led to a gradual decrease in particle size (Fig. 2a1–a4). This observation can be attributed to the appropriate amount of Ag assisting the anisotropic growth of gold particles along specific facets through UPD. The excess Ag uniformly covering the gold particle surface hinder the replacement of Ag with Au and the subsequent growth [32]. Gold mesostars synthesized under optimized conditions (0.5 mL AgNO_3_) exhibited a size of 1.016 ± 0.164 μm and a tip size of 520 ± 73 nm, as confirmed by the SEM images (Fig. 2b). In the UV–Vis–NIR absorption spectra of particles synthesized under each condition, larger particles exhibited a more gradual profile, which can be attributed to the broadening of the peaks beyond 1200 nm because of plasmon hybridization caused by an increase in particle size (Fig. 2c). We evaluated the SERS signals at the single-particle level to compare the extent of the local field enhancement by the particle morphology synthesized by controlling the Ag ions. The experiment was conducted using 1 µM Malachite Green isothiocyanate (MGITC) as a Raman reporter, and the SERS signals were compared as an average of 10 different particle measurements. The particles synthesized in 0.5 mL AgNO_3_ exhibited the strongest SERS signal attributed to the local field enhancement effect because these particles possess a mesoscale morphology with many sharp tips (Fig. 2d).

**Fig. 3:**
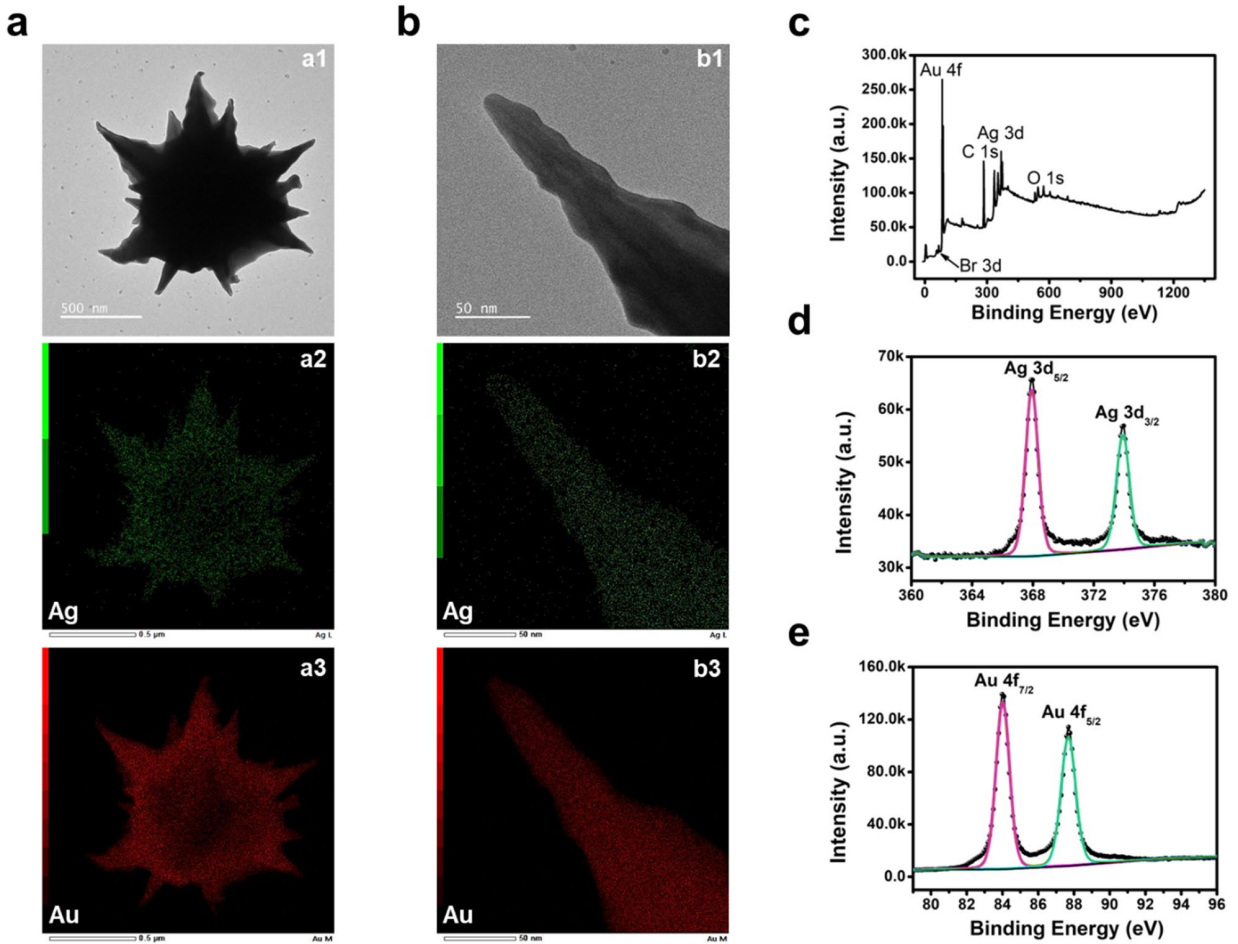
(**a**) TEM image of gold mesostar single particle (a1) Gold mesostar single particle with elemental mapping of (a2) Ag and (a3) Au. (**b**) TEM image of gold mesostar tip (b1) Gold mesostar tip with elemental mapping of (b2) Ag and (b3) Au. (**c**) XPS survey scan of gold mesostars. High-resolution XPS spectra of (**d**) Ag 3d and (**e**) Au 4f peaks

We analyzed the particle surface using elemental mapping and X-ray photoelectron spectroscopy (XPS) to verify the adsorption of Ag ions onto the gold surface through the AgUPD. Figure 3a and b show TEM and elemental mapping images for the entire particle and tip, respectively. The presence of Ag on the surfaces of both the gold mesostar and its tip was confirmed, indicating sublayer formation attributable to AgUPD. In the XPS survey scan, signals corresponding to Au and Ag from the gold mesostars, C and Br from CTAB, and O from the Si substrate were detected. The presence of Ag attributed to AgUPD on the gold mesostars was confirmed by peaks representing spin-orbit doublets at 368 and 374 eV for Ag 3d, whereas peaks at 84 and 87.6 eV for Au 4f_7/2_ and Au 4f_5/2_, respectively, were also observed (Fig. 3c and e).

**Fig. 4:**
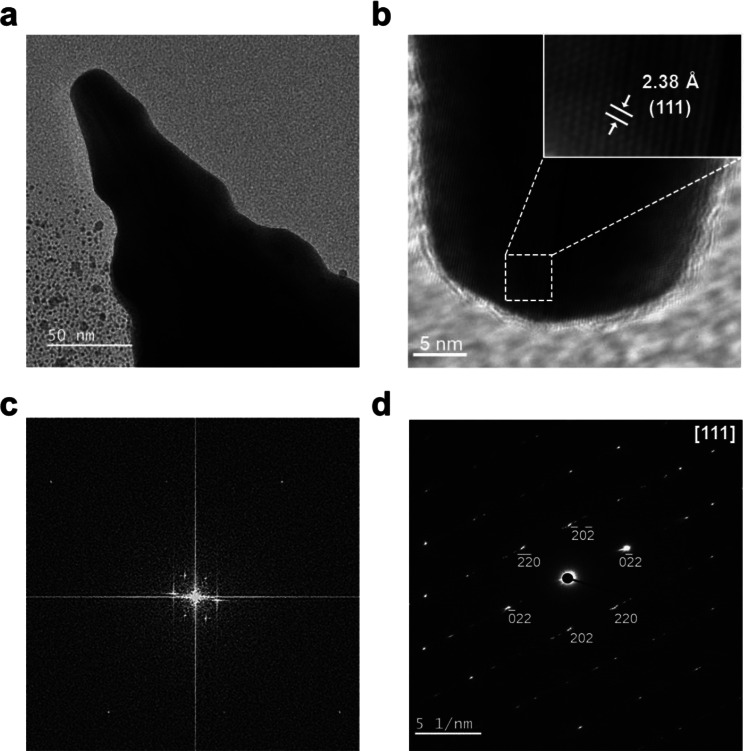
(**a**) TEM image of a gold mesostar tip. (**b**) HRTEM image of a branch tip (inter-plane spacing of 2.36 Å shows the presence of {111} planes.) (**c**) FFT pattern of a gold mesostar tip. (**d**) Hexagonal [111] SAED pattern of a gold mesostar tip

Lattice structures of the tips of the gold mesostars were verified using TEM, FFT, and SAED. The tips of the gold mesostars exhibited a d-spacing of 2.38 Å, which corresponds to an Au (111) plane (Fig. 4a and c). The hexagonal SAED pattern indicated a single-crystal structure at the tip, and indexing the diffraction spots confirmed the growth along the {111} facet (Fig. 4d). These results support the mechanism of anisotropic growth growth along the {111} facet caused by the previously described AgUPD, which inhibits growth along {110} facet.

**Fig. 5:**
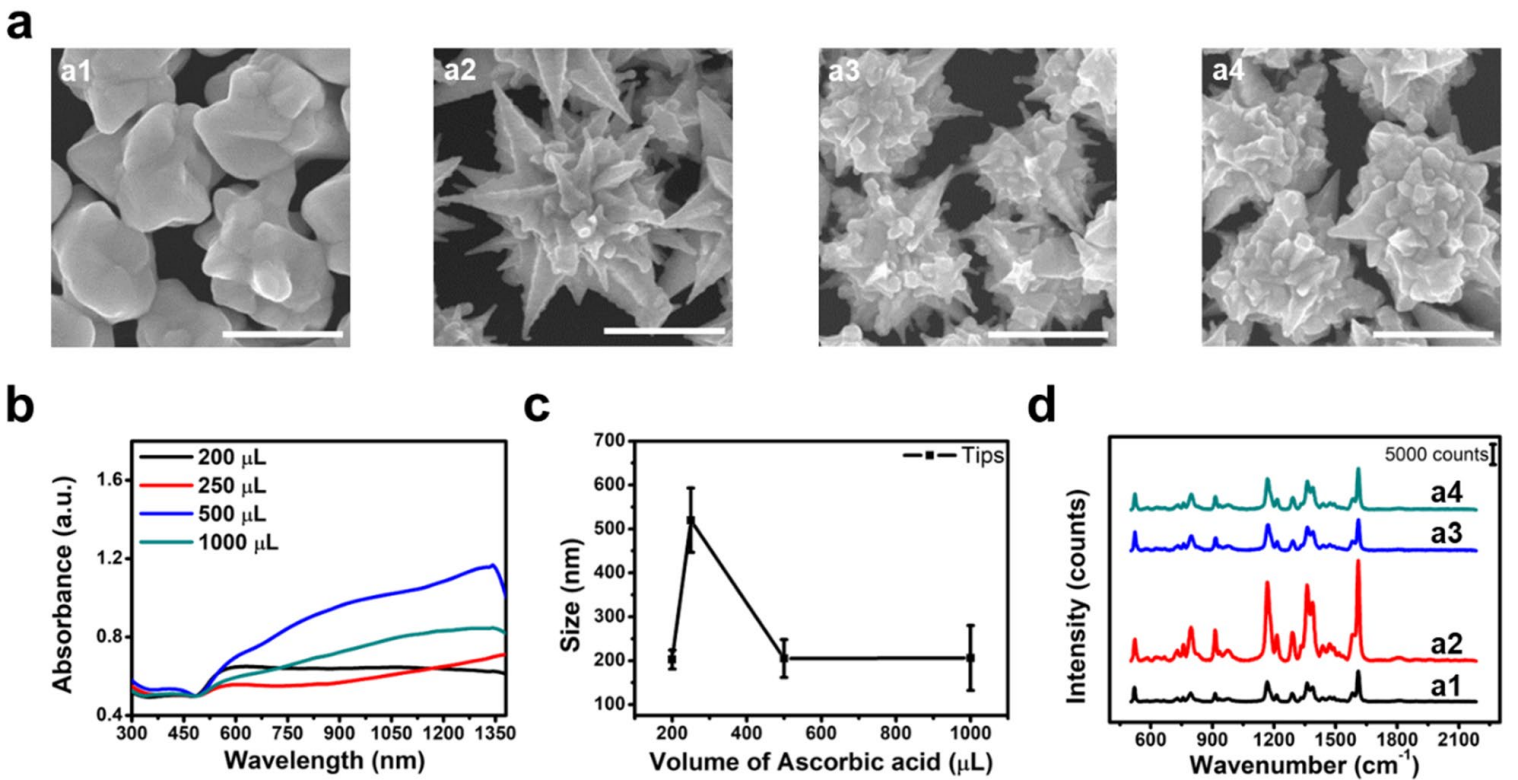
(**a**) SEM images of gold mesostars synthesized by adjusting the volume of AA in growth solution C (a1–a4 correspond to AA volumes of 200, 250, 500 and 1000 µL). All scale bars represent 1 μm. (**b**) UV-Vis-NIR spectra of gold mesostars synthesized under each condition. (**c**) Tip size of the gold mesostars. (**d**) SERS signals according to AA in growth solution C volume

LSPR is formed not only at the ends of the tips of gold mesostars but also at the bends of their sides, and therefore, obtaining sharp tips is crucial for generating strong SERS signals. The volume of ascorbic acid (AA) in the growth solution was adjusted to obtain sharp tips. Therefore, experiments were conducted using various AA volumes to investigate the effect of AA on tip formation. The synthesis of gold mesostars involves three sequential growth steps. Each growth solution (A, B, and C) contained AA as a reducing agent. The AA donates two electrons to the gold ions, reducing Au^3+^ to Au^+^ and forming the AuCl_2_^−^–CTAB complex [33]. Growth solution C contained a higher amount of AA than solutions A and B. As this is the stage where the final particle growth occurs, it directly affects the morphology of the gold mesostars. Figure 5a shows SEM images of the particles synthesized by adjusting the volume of AA in growth solution C. An insufficient AA volume leads to the unsaturated reduction of gold during the growth of gold mesostars, thereby preventing the formation of tips and hindering their growth (Fig. 5a1). In the case of excess AA, the electron transfer from the AA increases the negative charge on the surface of the growing gold particles, which weakens the UPD of Ag on the Au {110} facet, resulting in relatively isotropic growth and preventing the proper growth of tips (Fig. 5a3, a4) [34]. The UV-Vis-NIR absorption spectrum shows that the gold mesostars synthesized under conditions with AA > 0.5 mL exhibit a broad absorption peak in the visible region because the excess AA shortens the tip length of the gold mesostars and causes them to grow in an isotropic shape (Fig. 5b). The optimized conditions resulted in the synthesis of gold mesostars with the largest tip size of 520 ± 73 nm (Fig. 5c). We measured the SERS signals at the single-particle level using particles synthesized by controlling the volume of AA to compare the SERS signals based on the tip geometry. The longest and sharpest tips exhibited the highest SERS signals when AA was 0.25 mL (Fig. 5d). We adjusted the volume of AA in growth solutions A and B to precisely control the tip curvature. Growth solutions A and B contained a small amount of AA compared to C; however, they had a large effect on the tip curvature of the gold mesostars. We adjusted the facets of the seed particles to have sharper tips by adding an appropriate volume of AA to growth solutions A and B. Further, we found that the tip radius of curvature becomes smaller up to a certain volume (25 µL), whereas it becomes larger when an excessive amount of AA is added. The tip radius of curvature was less than 20 nm under optimized conditions. The SERS signals were the strongest when the tip curvature was the smallest (Fig. S1).

**Fig. 6:**
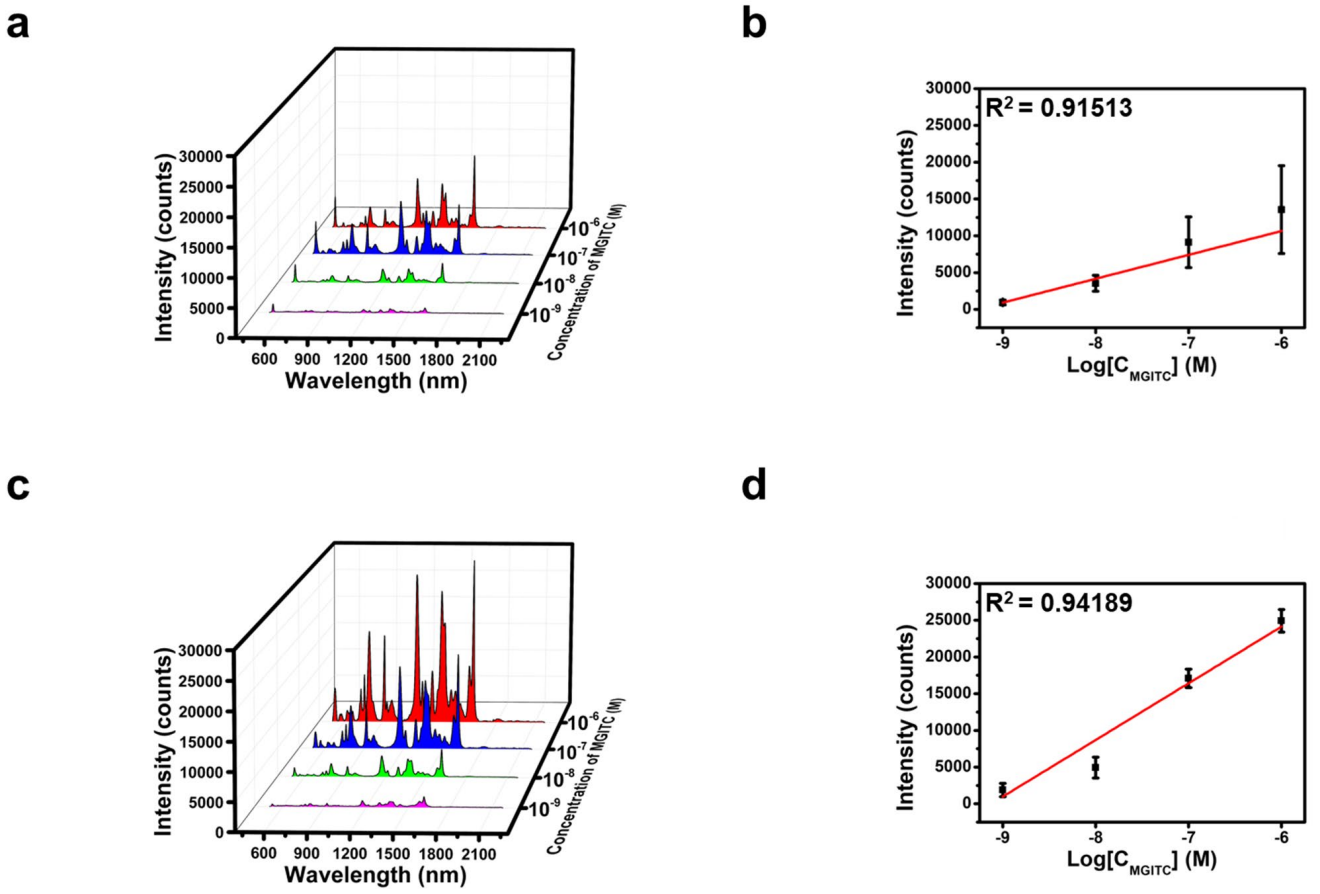
Comparison of SERS signals according to the concentration of MGITC (10^− 6^–10^− 9^ M) measured with a single gold mesostar (**a**) and a gold mesostars substrate (**c**). Calibration curve of the Raman intensity at 1616 cm^− 1^ versus the concentration of MGITC of the single particle (**b**) and gold mesostars substrate (**d**)

The high density of electrons concentrated at the tips on the surface of the gold mesostars formed localized fields that exhibited a strong SERS effect, even for single particles. We compared the SERS signals with the concentration of the Raman reporter (MGITC) to verify the SERS effect of a single gold mesostar synthesized under the optimized conditions. MGITC was detected even at an ultralow concentration of 10^–9^ M, and the R^2^ value of the concentration-dependent calibration curve at 1615 cm^–1^, one of the main peaks of MGITC, was 0.91513 (Fig. 6a and b). This result suggests that sensitive molecular detection can be achieved at the single-particle level.

We fabricated a SERS-active substrate by arranging these particles on a substrate to leverage the strong SERS effect of the gold mesostars. Fig. S2a shows the SERS-active substrate with uniformly and densely arranged particles. The SERS-active substrate exhibited enhanced SERS signals because of the local field enhancement effect caused by the tips of the particles, as well as the hot spots formed in the gaps between the particles. The fabricated substrate exhibited higher SERS signals than those of a single particle, with the signal intensity scaling with the concentration of MGITC. In addition, we confirmed that the R^2^ value increased to 0.94189 and the error bar decreased, thereby indicating that the fabricated SERS-active substrate produced a uniform signal with improved accuracy (Fig. 6c and d). Fig. S2b shows that the signal intensity of the Raman mapping image had a similar distribution, further corroborating the uniformity of the SERS signals. Furthermore, the Raman signal exhibited a low relative standard deviation value of 9.45% when 50 random spots were measured, thereby indicating uniform and sensitive SERS signals (Fig. S2c). These results suggest that SERS-active substrates prepared with gold mesostars exhibit high SERS efficiencies and have the potential to enable accurate and sensitive molecular detection.

## Conclusion

In this study, homogeneous gold mesostars were synthesized successfully using a three-step seed-mediated growth method. The resulting mesoscale particles with numerous sharp tips were used to fabricate SERS-active substrates for molecular detection. Anisotropic growth and formation of multifaceted mesostars were achieved by promoting multi-directional growth using Ag ions. The experimental investigations confirmed that the tip curvature could be precisely controlled by adjusting the amount of AA, which is a reducing agent. The gold mesostars exhibited a strong SERS effect, even at the single-particle level, because of the localized fields formed by the high density of electrons concentrated on the sharp tips. Upon fabricating SERS-active substrates by arranging these particles, a substantial enhancement in the electromagnetic field amplification was observed because of the formation of hotspots in the interparticle gaps. The SERS-active substrate showed enhanced SERS signals compared to that of a single particle, and a uniform and dense arrangement resulted in an even signal intensity distribution. These findings indicate that the fabricated SERS-active substrates can serve as effective sensing platforms for sensitive and accurate molecular detection.

### Electronic supplementary material

Below is the link to the electronic supplementary material.

Supplementary Material 1

## Data Availability

Not applicable.
