# Supplementary material for: Monodispersed mesoscopic star-shaped gold particles via silver-ion-assisted multi-directional growth for highly sensitive SERS-active substrates
Source: Nano Converg. 2024 Jul 4;11:26. doi: 10.1186/s40580-024-00435-4 (PMC11224182; doi:10.1186/s40580-024-00435-4)
Supplement: Supplementary file 1 — Supplementary Material 1 [file 40580_2024_435_MOESM1_ESM.docx]

**Supporting Information**

**Monodispersed Mesoscopic Star-Shaped Gold Particles via Silver Ion-assisted Multi-directional Growth for Highly Sensitive SERS-active Substrates**

Sumin Kim^1, 3, †^, Sunghoon Yoo^1, 3, †^, Dong Hwan Nam^1, 3^, Hayoung Kim^1, 3^, Jason H. Hafner^4^, Seunghyun Lee^1, 2, 3, *^

^1^ Department of Applied Chemistry, Hanyang University ERICA, Ansan, 15588, Republic of

Korea

^2^ Department of Chemical and Molecular Engineering, Hanyang University ERICA, Ansan,

15588, Republic of Korea

^3^ Center for Bionano Intelligence Education and Research, Hanyang University ERICA,

Ansan, 15588, Republic of Korea

^4^ Department of Physics and Astronomy, Rice University, 6100 Main St, Houston, TX 77005-1827, United States


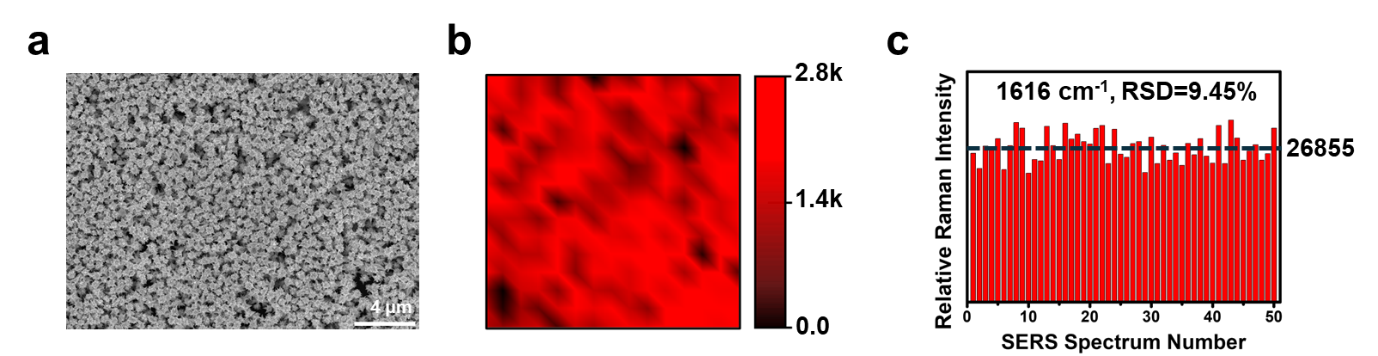

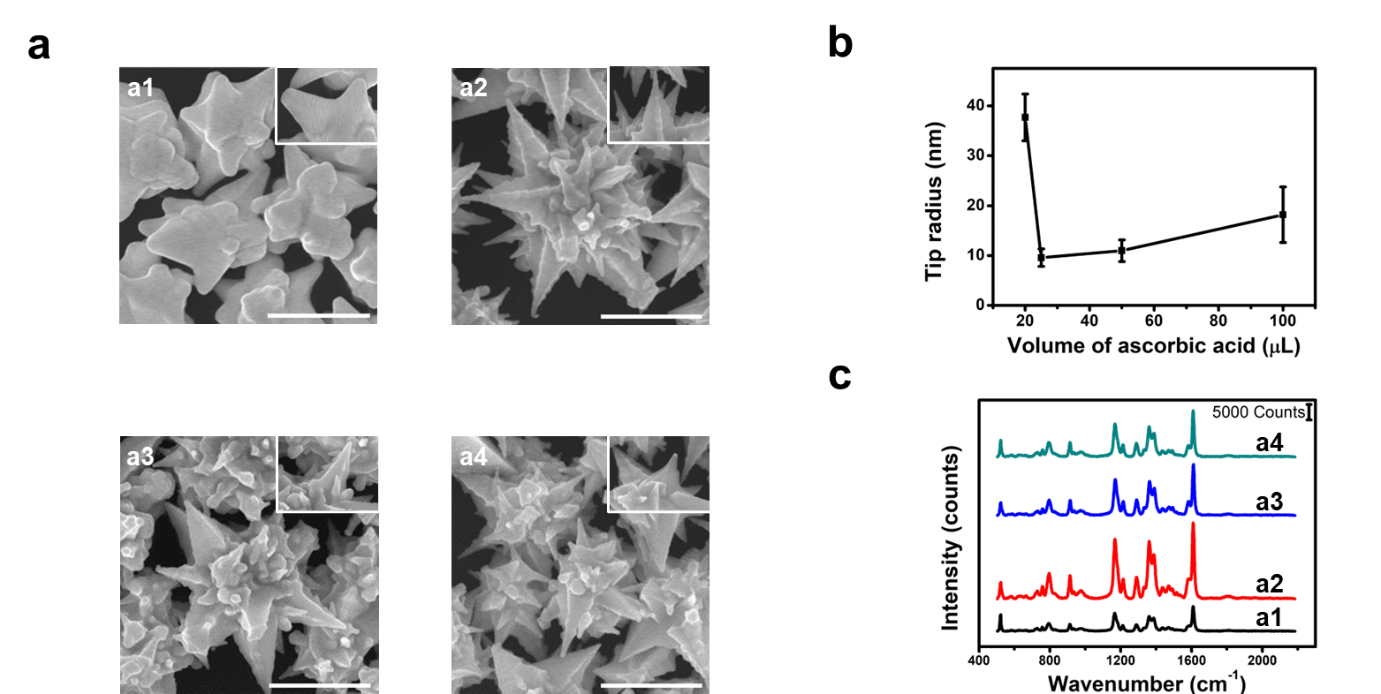


Fig. S2. (a) SEM image of the tip of a gold meso-stars substrate. (b) Raman mapping image measured on a 30 μm × 30 μm area of the SERS substrate. (c) RSD for the Raman peak intensities at 1616 cm^−1^. The data are based on the measurement of MGITC at 10^−6^ M.

Fig. S1. (a) SEM images of the tip of gold meso-stars synthesized by adjusting the volume of AA in growth solutions A and B (a1–a4 correspond to AA volumes of 20, 25, 50 and 100 μL). All scale bars represent 1 μm. (b) Corresponding tip radius of curvature. (c) SERS signals according to AA in growth solution A and B volume.
